# Parent’s Lived Experience of Memory Making With Their Child at or Near End of Life

**DOI:** 10.1177/10499091211047838

**Published:** 2021-09-16

**Authors:** Terrie Clarke, Michael Connolly

**Affiliations:** 1 Bayada Home Health Care, Dublin, Ireland; 2 UCD School of Nursing, Midwifery & Health Systems, University College Dublin, Ireland

**Keywords:** memory making, end of life, lived experience

## Abstract

**Background:**

Memory making is the process of creating mementos of a child with a life limiting condition, who may be at or near end of life, providing a tangible and visual connection to the child who has died.

**Aim:**

This study explored the lived experience a memory making process had on parents of children who were at or near end-of-life.

**Design:**

A qualitative approach was used. Hermeneutic phenomenology methods provided guidance to the data collection, with a more limited interpretative phenomenological analysis conducted.

**Setting:**

A purposive selected sample of 6 parents whose child had died and who had engaged in memory making participate. The sample was drawn from parents whose child had received care from a children’s hospice.

**Results:**

Individual interviews were conducted with 6 parents, all mothers. Three main themes emerged: Making the memories; the impact of memory making; and the end-of-life care journey. Parents experienced an overwhelmingly positive impact from memory making, as well as tangible and precious mementos that were created. The positive impact the process had on coping with grief and loss was also demonstrated, as well as the effect of helping to keep the deceased child’s memory alive and include them in conversation.

**Conclusions:**

The importance of skilled and sensitive staff with the ability to introduce the concept of memory making, and choice at end of life were highlighted by the parents who took part. Clinicians may benefit from understanding how memory making can positively impact the bereavement experience of parents whose child has died.

## Introduction

The death of a child or infant can have a detrimental impact on grieving parents and can lead to several adverse outcomes. Children with life limiting conditions encompass a range of different diagnoses including progressive conditions without curative treatment, irreversible but non-progressive conditions and life-threatening conditions such as cancer.^1^ While chronic illness is a prevalent cause of child mortality,^2^ there is limited, data available regarding the numbers of children with life limiting conditions globally at best it is underestimated. In Ireland, the number of children with a life limiting condition was estimated to be less than 400,^3^ however, this figure has been rapidly revised upward and the number of children in this category is now thought to be around 4,000 although this is possibly still under representing the actual total figure.^4^ Appropriate bereavement supports to meet the needs and prevent adverse outcomes for the proliferation of grieving parents with children who have life limiting conditions is evident.^5,6^

Memory making practices are increasingly recognized as a means of addressing this need for parents and their families.^7,8^ Memory making is the creation of individual pieces of art with families, capturing the participation of family members and significant people in the child’s life, and provides a tangible and visual connection which families treasure especially after the child has died.^9,10^ Fingerprint pendant projects,^11^ memory boxes or the retention of a lock of hair^9^ are examples of the type of activities that parents and guardians participate in. In essence, memory making projects have been found to aid those grieving the death of a child. Much of the evidence from pediatric studies originated in North America, while a number of major studies were carried out by the same team.^8,10,12-14^

## Background

The emergence of children’s palliative care was in response to the increase in and recognition of the number of children living with a life limiting condition.^15^ A life limiting condition of childhood is defined as:

A disease or illness for which there is no reasonable hope of a cure and of which the child will die from before the age of eighteen.^16^

The diagnosis of a life limiting or life-threatening condition has a profound and lasting effect on families and poses a huge challenge to lead a normal family life.^17^ The process and adjustment of grieving can be very individual and does not happen in isolation but rather in an environment where others are also grieving for the deceased. It happens at different levels affecting well-being and ability to cope, and can damage relationships and the family dynamic, with men and women differing in how they handle their grief.^18,19^ The loss of the child encompasses an entire journey into a new life, rather than being a single event.^20^ A 10-year ethnographic study of bereaved parents examined the consolation and resolution of their grief and findings clearly show how parents’ lives change following their loss. They subsequently live in a different world than before rather than returning to their previous life, with the growth and adaptation of their changed lives leading to resolution of their grief.^21,22^

Significant grief and adjustment difficulties are experienced by parents and siblings, and can last for years after their child’s death.^23^ Additionally, families have to deal with the burden of additional sources of stress which can lead to problems such as anxiety and distress.^18,19^ Re-establishment of a healthy family life can be very challenging, however with professional support parents and siblings can be enabled to connect both past and future in a beneficial way by creating communities of memory around their loved one.^24^

Memory making, also described as Legacy making may facilitate families to continue the bond with their child who has died in an inspiring and comforting way.^10,25^ Legacy is also defined in the literature as involving both family and patient to develop a meaning based coping system through the creation of a lasting memento,^26^ and is recognized as a source of inspiration, and is both important and beneficial to children and their families.^27^ Photographs and physical remembrances, such as a lock of hair or a handprint demonstrate tangible evidence of the child’s life, and as well as their physical belongings bring comfort to grieving parents.^6,28-30^

Addressing the bereavement support needs of parents at end of life is a fundamental step in identifying specific needs and providing appropriate support. In their study Tan et al^29^ selected infants with 3 complex and life-threatening conditions, with a complex clinical course and high rate of mortality. A series of digitally recorded narrative styled interviews were used to capture data from at least one parent per case to explore the emotion, anxieties and grief of the parents. Preparation and support for parents in the anticipatory grief stage leads to a smoother transition from being the provider of their child’s care to dealing with the death of their child and its aftermath and has a major impact on their grieving experience.^29^ The documented findings highlighted the importance and value of memory making in which tangible mementos formed evidence of the existence of their baby who may have never been brought home and integrated into family memories. Anecdotal evidence from an innovative precious print project in which a pendant was created imprinted with a fingerprint of the child demonstrated consistent appreciation and was reported as cherished and meaningful from the families who expressed gratitude that this project was available and they could partake in it.^31^

These findings are similar in a more recent qualitative study by Baughcum et al.^32^ Their aim was to improve end of life care in the neonatal intensive care setting, examined parents’ perspectives of their experiences of end of life with their infant, and captured a full range of parental experiences. Over 93% of parents reported they felt included in end-of-life decisions, guided by medical professionals. They also indicated a strong preference to have access to their baby and be in close proximity to them. Parents who were less involved in their infant’s bedside care reported this as a source of regret post bereavement. Participants of this study also valued the memory making activities which included the creation of personalized memory boxes, hand and foot print jewelry and moulds which document the infant’s life and assists parents to remain connected to the baby after death,^9,30,33^ and are a huge step in the grieving process.^20^

## Method

Memory making at end of life or shortly after death is facilitated in a children’s hospice service, and is often the last meaningful family activity that is experienced. When a child was referred to the service, and depending on the prognosis the team would offer the memory making service. Initial contact with family members was made by a member of the nursing team, where the memory making service was explained. Family members were shown examples of work that had be done with other families and this helped to plan their own memory making. Normally memory making occurred over a number of visits, with the majority of sessions taking place in the child’s own home, however in some instances sessions were conducted in the hospital due to the condition of the child at that time.

This study sought to explore the lived experiences of parents who had engaged in the memory making process at or near the point of their child’s death. Hermeneutic phenomenology using 3 analytic steps following Van Manen’s approach were to analyze the data through the art of writing and re-writings (Table 1). Phenomenology has been described as both a philosophical and methodological approach to research, which can be applied to a broad range of clinical research investigations in nursing.^34^ Therefore, this method was deemed most appropriate by the researcher in addressing the aim of this study.

**Table 1.** Data Analysis Steps (van Manen, 2016).^35^

Steps	Description of the steps	Interpretation of steps
First Step	Turning to a phenomenon of interest	Turning to the nature of the lived experience Expressing reflectively to make sense of the meaningful experience
Second Step	Conducting an investigation of the phenomenon	Develop a deep understanding of the phenomena as we live it rather than what we conceptualize it to be
Third Step	Reflecting on essential themes	Develop a deep understanding of the phenomenon through listening to, transcribing and reflecting and immersing in the data.

Purposive sampling was used for this study, with participants drawn from parents of children cared for in a hospice that provides a range of care services to children aged between 0-18 years who have been diagnosed with a life threatening or life limiting condition with palliative care needs. The sample was made up of parents who participated in memory making at or near their child’s death (Table 2).

**Table 2.** Sample Demographic.

Participant	Age of child at time of death	Place of death	Year of death	Memory making achieved/ facilitated
Participant 1	40 days	Home	2016	Days before death
Participant 2	21 months	Home	2017	Days before death
Participant 3	3 days	Home	2017	Day after death
Participant 4	22 months	Hospital	2017	Day after death
Participant 5	22 days	Hospital	2017	Day after death
Participant 6	10 years	Hospice	2016	Days before death

Prior to the commencement of this study a “gatekeeper” was identified to assist and identify individuals suitable to recruit as participants. Potential participants were identified from the bereavement database of the organization and the gatekeeper sent the initial invitation to those identified to participate by post along with the details of the researcher. Based on the inclusion criteria and permitted by the ethics committee parents whose child had died in the last 12 months were not contacted.

Ethical permission to conduct the study was provided by Research and Ethics Committee of the children’s hospice (LLEC1 15/01/2019) and exemption from ethical approval was granted by the University College Dublin School of Nursing, Midwifery & Health Systems (REC 18046).

### Data Collection

Data was collected by audio-recorded individual interviews with participants who had given written consent to participate. An interview schedule was used for the interviews to facilitate the exploration of topics of relevance (Table 3). It was important that the in-depth interviews were conducted in a quiet, private environment without interruption to enable participants to talk freely about their very personal experiences regarding the death of their child and the subsequent impact of the memory making process. Participants were given a choice of location for the interview and all except one participant opted to complete the interview in their own home. That one interview was conducted in the hospice. Given the sensitive nature of the content for the interview, the interviewer ensured that clarification was given before the interview commenced regarding the purpose of the interaction and the context and the use of a digital recorder. Any questions or concerns were addressed.

**Table 3.** Interview Schedule.

Tell me about your child and your experience of Memory Making? Tell me how this made you feel? Did other family members participate, and can you tell me how that made you feel? Tell me how supported you felt during this process? Have you any suggestions or comments that can help to develop the service further? Is there anything else you would like to add?

### Data Analysis

The process of data collection and data analysis occurred concurrently following the commencement of the interview with the first participant.^36^ All verbatim transcripts of interviews were checked for accuracy by both researchers.

The influence of a phenomenological approach, and specifically Van Manen’s approach formed the basis of the analysis. The thematic analysis involved the use of Van Manen’s 3 approaches: (i) the holistic or sententious approach, (ii) the selective or highlighting approach, and (3) the detailed or line-by-line approach. This process helped to identify and isolate key thematic aspects of the study phenomenon and prompts used at each of the individual interviews.

A selective data analysis technique was used as part of the initial data analysis, where the verbatim transcripts were read several times to highlight the core statement(s) or phrase(s) that appeared to be essential to the description of the phenomenon or experience being described by the parents.^37,38^ This holistic approach considered each interview transcript as a whole, expressing the meaning of the experience of the mothers by formulating a phrase to describe their experience. Listening to the recording while reading the transcript several times and continually asking what statement or phrase was particularly essential to revealing the human phenomenon or the experience being described.^37,38^ Finally, in a detailed approach, we aimed to capture the meaning of the experience of memory making for their child who had died, portrayed as their life stories.

A reflective journal of notes after each interview, field notes taken during and after the individual interviews were also considered during the data analysis. While the use of software for data analysis was considered the researchers opted to manually analyze and organize the data collected.^37^

## Results

Six bereaved parents responded to the invitation to participant and agreed to take in an audio-recorded interview for the study (Table 3). Although mothers and fathers were invited to take part, only mothers took part in the interviews. Of the 6 interviewed, 3 were mothers of a child who had died at home; 2 were the mother of child who died in hospital and 1 mother’s child had died in a hospice. The children who had died ranged in age from 3 days to 10 years. For half of those interviewed memory making was achieved before the day of death, while for the other half memory making occurred after the death had occurred.

Through iterative analysis based on Van Manen’s approach 3 main themes emerged from the data: Making the memories; the impact of memory making; and the end-of-life care journey (Table 4).

**Table 4.** Themes and Categories of Experience of Memory Making.

Theme	Categories
Making the memories	Introducing the concept of memory making Choice, guidance and support The activity of memory making Individuality of creations Participation of siblings in the activity
The impact now of the memory making.	Readiness to receive the memory making Impact of participating in memory making Future impact of memory making Impact of memory making on grieving the loss
The end of life care journey	Choice at end of life Alleviating fear of death Early referral to hospice services Gratitude for the opportunity to make precious memories

### Making the Memories

There was a general consensus among all participants about wanting to make the most of precious time and create cherished memories and keepsakes. The findings demonstrate the importance of introducing the concept of memory making to families, who are often at a very difficult stage with their child’s illness. Although families often do not understand or have never considered the concept of what memory making is or what the process involves, many expressed gratitude that the subject was broached, and the opportunity afforded them to create memories and tangible pieces of art.

One mother who received an antenatal diagnosis and whose baby died at 9 days of age, spoke about beginning the memory making process during her pregnancy and conveyed the significance of memory making for her:

I just wanted to make as many memories as I could and I thought it was, you know, if there was someone to come and help me to do that then great (P3)

Another mother expressed her feelings about memory making during her baby’s final days:

We wanted to have as much memories as we could have (P1)

Another mother conveyed her feelings and the importance of memory making to her family:

…made some lovely memories…That’s something that was very important for us (P6)

The activities of the memory making, and the mementos created were individual to each family and situation, and included hand and foot moulds, hand and footprints, fingerprint decorations. One mother explained how she and her family chose what they would like to do, and how it was important to have input into the process:

I think it was mostly all of our input (P2),

She spoke about creating precious white footprints on black paper each with a set of their prints and their dying daughter’s which were later framed:

The girls who came from [name of care facility] gave us a book [pause] of kind of previous examples…Kind of picked out what we would like…We got to kind of [pause] pick a piece [pause] that was kinda special for each of us…. that we could keep for ourselves…something that would be special to each of us (P2)

Taking part in the process was seen as a very important part of the experience for parents. Another mother who participated in memory making in the neonatal unit after her son’s death shared her experience of being able to participate in her son’s care after his death. This followed 3 weeks of not being able to because of the barriers of a neonatal intensive care environment such as tubes, ventilator and incubator:

Tom [Baby’s father] and I emm [pause] got to help with Patrick like we never [pause] got to wash him or bathe him [pause] or do anything with him until he died [pause] we didn’t get to hold him or hug him and kiss him (P5).

She continues recounting this precious and valuable time for them both spent making memories with their baby after he had passed away:

For us to you know, take part [pause] you know in making his footprints and putting paint on him was heart breaking [pause] but it was massively important to us to be part of it rather than somebody else doing it and taking him away to do it (P5).

### The Impact of the Memory Making

When asked to recount the day they received the memory making creations many feelings and emotions were expressed. Overall parents were underprepared for how overwhelming the experience of receiving these items was for them, however, they were also overjoyed to have these creations.

One mother (P3) who lost her baby at 9 days of age spoke about how she did not expect it to be as emotional as it was but spoke of the joy at receiving tangible pieces:

I didn’t think it would have [pause] the impact, I didn’t think that it would be as emotional (P3).

Another mother recounted the day and the fact that she hadn’t put much thought into it:

I had been like yeah, of course pop out (P4).

She then continued to discuss the impact of receiving these items:

I very suddenly realized what was happening and that this, you know, this was the first time I was going to see this stuff (P4).

The joy and the impact of receiving these creations was evident in all of the participants.

Describing the memory making in her home one mother said:

This little girl is there and [pause] if I didn’t have those…I’d find it very difficult because they give me so much comfort (P3).

Similarly, another mum spoke about the memory making, and in particular foot and hand moulds:

They’re just more tangible, they’re more real [pause] you can feel them and remember (P5)

She also spoke about the significance for her of a family tree hanging in her hallway. It communicates an invitation to mention her baby son, appreciating that though it is difficult for people it is welcomed, and hurts when people don’t talk about him:

They’re given an invitation to mention him and that’s welcomed…It hurts when people don’t (P5)

All participants spoke positively about the impact of having the tangible memories of their children and the impact this has on their grief, and how it helps and comforts them to deal with their loss. One mother said with regard to this:

The memory making I suppose [pause]…it kind of saved us a lot…For every important event we make memories now (P4).

### The End-of-Life Care Journey

The location for end-of-life care and being able to choose this was important for a number of families. One mother spoke of the realization in hospital that her baby’s time was drawing close talked of her baby letting them know she was not going to be here for long:

She was letting us know that she wasn’t going to be here forever, so we just had this urge just to get her home (P3).

Another mother spoke of the limitations of being in hospital at end of life and how not all of the family could go to visit the baby in intensive care:

We took Emma home because we didn’t really want to be in hospital…we wanted all out family to meet Emma and say goodbye to her…It was great to have everyone come and make memories that day (P1).

Another mother (P2), whose daughter died from cancer spoke of her wish that her daughter had been referred to the children’s hospice sooner in her illness. She feels earlier referral would have facilitated the creation of more memories and that her daughter would have been able to participate in these and enjoy them:

If maybe we had been referred sooner [pause] we would have had a chance to do that [memory making sessions at home with her child participating] (P2).

She acknowledged the difficulties of having that conversation when they were still hoping for a cure:

Does it tell a parent that they [medical team] have actually given up on their child coming through this [cancer] (P2).

However, she still feels the conversations need to be held earlier saying:

It wouldn’t have changed the outcome for all of this. She still would have passed away but we would have had an even more enhanced memory of the memory making that we do now (P2).

These are worthy issues for healthcare staff to consider and give some credibility to when planning the care of families facing the loss of their child, in order to guide support and navigate their journey with them.

## Discussion

This study sought to explore the lived experiences of parents who participated in memory making with their child at or near end of life. The findings demonstrate the importance and the willingness of parents to have the opportunity to create special and precious mementos with their child when time is short. It is recognized that caring for a child with palliative needs involves the whole family,^5,6,33,39^ and that challenges exist in providing effective and appropriate palliative care and support for children with complex health needs.^14,32,40,41^

The findings from this study indicated that all of the families interviewed grasped the opportunity to make precious memories with their child, realized how time was short, and believed this experience would facilitate the bond for the family with the child who had died. One mother described how she began making memories in pregnancy when she discovered her baby’s life limiting diagnosis as each precious day was an achievement for her family. These findings mirror those of Terrah et al^12^ who documented similar desires of bereaved families in maintaining a bond with their child in her research.

Introducing the concept of memory making requires skill and sensitivity from experienced nurses, and in a timely manner. However, in the context of the participants of this study there was not always the time available to develop this relationship, therefore skill, understanding and sensitivity was fundamental to introducing the concept to families. The role of professionals in caring for families at or close to end of life was also highlighted as many of those participants in this study spoke about how they would not have thought of doing any memory making activities with their child at that time.

The emotional distress and grief that were present during this time was paramount, and almost all the participants stated they would not have thought of doing it, and were grateful for staff who introduced the concept of memory making activities to them. Raymond et al^42^ identified the great importance of memory making as part of the provision of end-of-life care, and it has also been depicted as a means to help grieving parents to develop a coping system through the creation of lasting memories and mementos.^26,28,32,43^

These findings are similar to those of Tan et al^29^ who documented that the process of memory making provided the opportunity for precious and valuable time as a family together.

While the findings showed an overall positive impact and delight to have such treasured and special reminders of their child, it was evident that each family had their own ways of dealing with their grief and their journeys while similar in ways were individual to each family. These personalized creations provided an avenue for parents to remain connected with their loved one after death, similar to findings of a study conducted on neonatal palliative and end-of-life care in England by Branchett and Stretton.^33^

There was a general recognition among all participants of how impactful the receiving of the memory making creations was and all participants said it was much more emotional and powerful than they had anticipated, highlighting the importance of fully preparing families for receiving the memory making creations. The families took possession of these items, from the hospice, in the weeks and months after their child’s death when they felt ready for this, and this varied for each participant with some families anxious to see their creations very soon after the death of the child. In some instances the time between the death and receipt of the creations was due simply to the time it took for moulded creations to be complete and mounted into frames. Others took months to be able to contemplate being ready to receive it, due to their emotional readiness to see the created memories. A number of those interviewed revealed how they felt they had underprepared themselves for the emotional impact of seeing the items for the first time.

All parents referred to the fact that the tangible momentos helped hugely in their loss and grief. Being able to touch and feel the moulds was extremely comforting for 4 of the families. Three participants referred to these items as creating a bond with their deceased child, which would last well into their future life. One participant spoke about how these items help her on the “dark days.” Continuing bonds are essential and fostered and developed over time following the death of a loved.^5,6,42,44^ and interact with meaning making in response to that loss.^43^ Re-establishing a healthy family life can be challenging after the loss of a child,^24^ however those who made sense of their loss in a way which was meaningful had less associated symptoms of complicated grief.^32,43^

All participants appeared ready and able to speak about their child’s end of life experience, though it was emotional at times. End-of-life care for children can be fraught with complex legal and ethical dilemmas as well as emotions, and evidence is scarce and lagging behind the adult cohort on the provision of optimal end-of-life care for children.^45^ Death and especially the death of a child can be an extremely frightening experience for families. Findings from previous studies demonstrate the huge benefit of cherished and unrestricted time spent with the child after death, and the opportunity to perform caregiving activities. Findings also demonstrated the bond between the child and parents may be strengthened by ensuring parents have maximum access to their child, spending valuable private time together, and receiving precious keepsakes.^28,32^

## Conclusion

The findings from this study provide insight into this meaningful and worthwhile activity in the context of children’s palliative care in an Irish setting. This study has highlighted the great benefits and satisfaction experienced from the provision of a memory making service in a children’s hospice. It has also highlighted the skills required in order to provide this service in a compassionate and sensitive manner during times of great distress for families. There can be no doubt that the findings of this study offer theoretical evidence as to the overall positive and welcome experience for the families as well as the perception of enhanced end of life and bereavement care and support. Further research on this topic will enhance and confirm the importance of the memory making activity as an essential component in children’s palliative care, and further investigation and exploration of this concept will lead to improvements in the service provided and the experiences of children, families and the professionals involved.

### Limitations of the Study

Limitations to this study are identified. The first limitation relates to the sample size. While the findings could not be generalized it is common that qualitative and phenomenological studies may contain a lower number of participants. This is acceptable as the purpose of the study is not to obtain generalized findings but those specific to the area of inquiry, including the common and shared experiences of the participants. Another limitation of this study relates to the site of the study. All of the participants were recruited from a single palliative care service, a children’s hospice. The findings though are relevant to and can be valuable for any service or department providing end-of-life care for children and families. A further limitation identified was that the principal researcher knew all of the participants in a professional capacity, who was involved in their care either during end of life, memory making, or the post bereavement visit to bring the finished memory making creations to the family. The impact of this on their decision to participate, the interview process and findings is difficult to determine. Although it is possible that the previous relationship enabled the participants to be comfortable, open and honest in recounting their experiences.

## References

[1] Department of Health and Children (DOHC). Palliative Care for Children with Life-Limiting Conditions in Ireland—A National Policy. Stationary Office; 2009.

[2] Modell B Berry RJ Boyle CA , et al. Global regional and national causes of child mortality. Lancet. 2012;380(9853):1556; author reply 1556-1557. 23122246 10.1016/S0140-6736(12)61878-9

[3] Department of Health and Children (DOHC) and the Irish Hospice Foundation (IHF). A Palliative Care Needs Assessment for Children. Stationery Office; 2005.

[4] Ling J O’Reilly M Balfe J Quinn C Devins M . Children with life limiting conditions: establishing accurate prevalence figures. Ir Med J. 2015;108(3):93. 25876306

[5] Dutta O Tan-Ho G Choo PY , et al. Trauma to transformation: the lived experience of bereaved parents of children with chronic life-threatening illnesses in Singapore. BMC Palliat Care. 2020;19(1):46. 32252753 10.1186/s12904-020-00555-8 PMC7137327

[6] Thornton R Nicholson P Harms L . Creating evidence: findings from a grounded theory of memory-making in neonatal bereavement care in Australia. J Pediatr Nurs. 2020;53:29–35. 32344367 10.1016/j.pedn.2020.04.006

[7] Akard TF Duffy M Hord A , et al. Bereaved mothers’ and fathers’ perceptions of a legacy intervention for parents of infants in the NICU. J Neonatal Perinatal Med. 2018;11(1):21–28. 29689746 10.3233/NPM-181732

[8] Schaefer MR Wagoner ST Young ME Madan-Swain A Barnett M Gray WN . Healing the hearts of bereaved parents: impact of legacy work on grief in pediatric oncology. J Pain Symptom Manage. 2020;60(4):790–800. 32360992 10.1016/j.jpainsymman.2020.04.018

[9] Carlson R . Helping families create keepsakes when a baby dies. IJCB. 2012;27(2):86–91.

[10] Foster TL Dietrich MS Friedman DL Gordon JE Gilmer MJ . National survey of children’s hospitals on legacy-making activities. J Palliat Med. 2012;15(5):573–578. 22577785 10.1089/jpm.2011.0447 PMC3353751

[11] Miller LH Lindley LC Mixer SJ Fornehed ML Niederhauser VP . Developing a perinatal memory-making program at a children’s hospital. *MCN Am* J Matern Child Nurs. 2014;39(2):102–106. 10.1097/NMC.0000000000000016 24566160

[12] Terrah L Foster T Gilmer MJ , et al. Bereaved parents’ and siblings’ reports of legacies created by children with cancer. J Pediatr Oncol Nurs. 2009;26(6):369–376. 20032298 10.1177/1043454209340322 PMC2835334

[13] Boles JC Jones MT . Legacy perceptions and interventions for adults and children receiving palliative care: a systematic review. Palliat Med. 2021;35(3):529–551. 33487090 10.1177/0269216321989565

[14] Akard TF Wray S Friedman DL , et al. Transforming a face-to-face legacy intervention to a web-based legacy intervention for children with advanced cancer. J Hosp Palliat Nurs. 2020;22(1):49–60. 31804281 10.1097/NJH.0000000000000614 PMC6940537

[15] Quinn C Bailey M . Caring for children and families in the community: experiences of Irish palliative care clinical nurse specialists. Int J Palliat Nurs. 2011;17(11):561–567. 22240634 10.12968/ijpn.2011.17.11.561

[16] ACT. A Guide to the Development of Children’s Palliative Care Services. Association for Children with Life-threatening or Terminal Conditions and Their Families; 2009.

[17] Eilertsen ME Eilegård A Steineck G Nyberg T Kreicbergs U . Impact of social support on bereaved siblings’ anxiety: a nationwide follow-up. J Pediatr Oncol Nurs. 2013;30(6):301–310. 24326219 10.1177/1043454213513838

[18] Stroebe M Schut H Boerner K . Continuing bonds in adaptation to bereavement: toward theoretical integration. Clin Psychol Rev. 2010;30(2):259–268. 20034720 10.1016/j.cpr.2009.11.007

[19] Stroebe M Schut H . The dual process model of coping with bereavement: a decade on. *Omega (Westport)*. 2010;61(4):273–289. 10.2190/OM.61.4.b 21058610

[20] Williams C Munson D Zupancic J Kirpalani H . Supporting bereaved parents: practical steps in providing compassionate perinatal and neonatal end-of-life care—a North American perspective. Semin Fetal Neonatal Med. 2008;13(5):335–340. 18472317 10.1016/j.siny.2008.03.005

[21] Klass D . Solace and immortality: bereaved parents’ continuing bond with their children. Death Stud. 1993;17(4):343–368.

[22] Pearson H . Managing the emotional aspects of end of life care for children and young people. Paediatr Nurs. 2010;22(7):31–35. 10.7748/paed2010.09.22.7.31.c7951 20954527

[23] Thompson A Miller K Barrera M , et al. A qualitative study of advice from bereaved parents and siblings. J Soc Work End-of-Life Palliat Care. 2011;7(2-3):153–172. 10.1080/15524256.2011.593153 PMC3230284 21895435

[24] Bugge KE Darbyshire P Røkholt EG Haugstvedt KT Helseth S . Young children’s grief: parents’ understanding and coping. Death Stud. 2014;38(1-5):36–43. 24521044 10.1080/07481187.2012.718037

[25] Foster T Gilmer MJ Bavies B , et al. Bereaved parents and siblings reports of legacies created by children with cancer. J Pediatr Oncol Nurs. 2009;26(6):369–376. 20032298 10.1177/1043454209340322 PMC2835334

[26] Allen R . The legacy project intervention to enhance meaningful family interactions: case examples. Clin Gerontol. 2009;32(2):164–176. 20046967 10.1080/07317110802677005 PMC2682362

[27] Akard TF Dietrich MS Friedman DL , et al. Digital storytelling: an innovative legacy-making intervention for children with cancer. Pediatr Blood Cancer. 2015;62(4):658–665. 25586983 10.1002/pbc.25337 PMC4339662

[28] Blood C Cacciatore J . Parental grief and memento mori photography: narrative, meaning, culture and context. Death Stud. 2014;38(1-5):224–233. 24524585 10.1080/07481187.2013.788584

[29] Tan JS Docherty SL Barfield R Brandon DH . Addressing parental bereavement support needs at the end of life for infants with complex chronic conditions. J Palliat Med. 2012:15(5):579–584. 22512806 10.1089/jpm.2011.0357 PMC3353765

[30] Mullen J Reynolds M Larson J . Caring for pediatric patients families at the end of the child’s life. Crit Care Nurse. 2015;35(6):46–55; quiz 56. 26628545 10.4037/ccn2015614

[31] Miller LH Mixer SJ Lindley LC Fornehed ML Niederhauser V Barnes L . Using partnerships to advance nursing practice and education: the precious prints project. J Prof Nurs. 2015;31(1):50–56. 25601245 10.1016/j.profnurs.2014.06.003

[32] Baughcum AE Fortney CA Winning AM , et al. Perspectives from bereaved parents on improving end of life care in the NICU. Clin Pract Pediatr Psychol. 2017;5(4):392–403.

[33] Branchett K Stretton J . Neonatal palliative and end of life care: what parents want from professionals? J Neonatal Nurs. 2012;18(20):40–44.

[34] Feeley C . Freebirthing: a case for using interpretive hermeneutic phenomenology in midwifery research for knowledge generation, dissemination and impact. J Res Nurs. 2019;24(1-2):9–19. 34394499 10.1177/1744987118809450 PMC7932453

[35] van Manen M . Researching Lived Experience: Human Science for an Action Sensitive Pedagogy. Taylor Francis; 2016.

[36] Streubert-Speziale H Carpenter DR . Qualitative Research in Nursing, Advancing the Humanistic Imperative. Lippincott Williams & Wilkins; 2011.

[37] Van Manen M . Phenomenology of Practice. Left Coast Press, Inc; 2014.

[38] Van Manen M . Researching Lived Experience: Human Science for an Action Sensitive Pedagogy. 1st ed. The Althouse Press; 2007.

[39] Polit DF Beck CT . Essentials of Nursing Research: Appraising Evidence for Nursing Practice. 8th ed. Wolters Kluwer Health/Lippincott Williams & Wilkins; 2014.

[40] Benini F Spizzichino M Trapanotto M Ferrante A . Pediatric palliative care. Ital J Pediatr. 2008;34(1):4. 19490656 10.1186/1824-7288-34-4 PMC2687538

[41] Vickers J Chrastek J . Place of care in Goldman A. In: Hain R Liben S , eds. Oxford Textbook of Palliative Care for Children. Oxford University Press; 2012.

[42] Raymond A Lee SF Bloomer MJ . Understanding the bereavement care roles of nurses within acute care: a systematic review. J Clin Nurs. 2017;26(13-14):1787–1800. 10.1111/jocn.13503 27504875

[43] Neimeyer R . Complicated grief and the reconstruction of meaning: conceptual and empirical contributions to a cognitive-constructivist model. Clin Psychol Rev. 2006;13(2):141–145.

[44] Packman W Horsley H Davies B Kramer R . Sibling bereavement and continuing bonds. Death Stud. 2006;30(9):817–841. 17004367 10.1080/07481180600886603

[45] Bergstraesser E Zimmermann K Eskola K Luck P Ramelet AS Cignacco E . Paediatric end-of-life care needs in Switzerland: current practices, and perspectives from parents and professionals. A study protocol. J Adv Nurs. 2015;71(8):1940–1947. 25740472 10.1111/jan.12650

